# Glycerol to Glyceraldehyde Oxidation Reaction Over Pt-Based Catalysts Under Base-Free Conditions

**DOI:** 10.3389/fchem.2019.00156

**Published:** 2019-03-27

**Authors:** Ayman El Roz, Pascal Fongarland, Franck Dumeignil, Mickael Capron

**Affiliations:** ^1^CNRS, Centrale Lille, ENSCL, Université d'Artois, UMR 8181-UCCS-Unité de Catalyse et Chimie du Solide, Université de Lille, Lille, France; ^2^Laboratoire de Génie des Procédés Catalytiques, Université Lyon 1, Villeurbanne, France

**Keywords:** glycerol, oxidation, base-free conditions, platinum, glyceraldehyde

## Abstract

Glycerol valorization through partial oxidation is a good way of obtaining many different molecules with high added value such as glyceric acid, tartronic acid, dihydroxyacetone, etc. Among the potential products, glyceraldehyde is an interesting chemical compound for its various applications in different domains such as organic chemistry, medical, and cosmetic industries. In the present paper, we studied the effect of different supports on the glycerol oxidation reaction in a batch reactor applying base-free conditions. The tested catalysts were Pt-based materials deposited on various supports (i.e., SiO_2_, TiO_2_, ZSM-5, γ-Al_2_O_3_), which were synthesized using a deposition method followed by a chemical reduction. The catalysts were extensively characterized (BET, ICP, XRD, TEM, XPS), highlighting differences in terms of specific surface areas, textural properties, and Pt nanoparticles sizes. We evidenced a direct relation between glycerol conversion and glyceraldehyde selectivity (i.e., an increase in glycerol conversion leads to a decrease in glyceraldehyde selectivity). The Pt/γ-Al_2_O_3_ catalysts exhibited the highest activity, but their selectivity to glyceraldehyde significantly decreased with time on stream. Pt/SiO_2_ presented the highest selectivity to glyceraldehyde owing to a slower reaction rate, which allows envisioning technical opportunities to continuously extract the formed glyceraldehyde from the mixture.

## Introduction

Biomass efficient valorization is an important challenge to ensure the viability of our various industrial sectors (Katryniok et al., 2011; Skrzynska et al., 2015). In fact, the increasing demand for energy, combined with the environmental issues related to climate change, motivates the promotion of the use of renewable energies and biofuels, as well as of the replacement of the fossil raw materials traditionally used in the chemical industry with bioresources (Purushothaman et al., 2014). In this context, biodiesel is obtained by catalytic transesterification of the vegetables oil or animal fat with short chain alcohols, mainly methanol (Ftouni et al., 2015). The main by-product of this reaction is glycerol (accounting for 10 wt.% based on the initial plant oil quantity). The valorization of this molecule has been intensively studied in several ways, including advanced transformations to value added compounds (Katryniok et al., 2011). Its upgrading by heterogeneous catalysis has been particularly studied, in gas phase to produce for instance acrolein (Katryniok et al., 2009, 2010; Lauriol-Garbey et al., 2011), acrylonitrile (Liebig et al., 2013) or H_2_ (El Doukkali et al., 2014) but also in liquid phase including conversion to esters or glycerol ethers by esterification or etherification, to *1,2*-propanediol or *1,3*-propanediol by hydrogenolysis (Alhanash et al., 2008). The glycerol partial oxidation in the liquid phase can lead to various products such as aldehydes (glyceraldehyde), ketones (dihydroxyacetone), and carboxylic acids (glyceric acid, tartronic acid, glycolic acid, etc.) (Katryniok et al., 2011; Mimura et al., 2014; Skrzynska et al., 2016, 2019; Zaid et al., 2017).

In this paper, we focus on the glycerol oxidation using noble metal catalysts to obtain glyceraldehyde in base free media. Glyceraldehyde is an industrially important chemical compound, which has found applications in the cosmetic industry, organic chemistry and, in pharmaceutical applications. The main difficulty lies in the control of the catalytic selectivity with many possible products through networks of consecutive reactions (Skrzynska et al., 2015; Díaz et al., 2017), which can also ultimately lead to low value products, such as oxalic acid or carbon dioxide. Until now, most of the heterogeneous catalysts used for the oxidation of glycerol are based on noble metals such as platinum, palladium, gold, or even silver (Carrettin et al., 2002, 2003; Zhang et al., 2012; Skrzynska et al., 2014; Ftouni et al., 2015). According to the various studies reported in the literature, it is clear that the gold-based catalysts are active only in basic solution. Under these conditions, glycerol oxidation over supported gold catalysts promotes the production of acids under their salt form, with the necessity of an additional step to recover the desired acids in their native form, yielding a high quantity of waste (i.e., salts resulting from the neutralization). The use of the Ag based catalyst, in basic media, leads to the formation of glycolic acid resulting in an oxidative C-C bond cleavage. On the other hand, the platinum based catalysts enable the conversion of glycerol in both acidic and basic solutions and can be selective for the formation of glyceraldehyde in acidic solution or base-free solutions (Carrettin et al., 2002, 2003; Zhang et al., 2012; Skrzynska et al., 2014; Wang et al., 2014; Li and Zaera, 2015). In these previous studies, authors evidence that selectivity is a critical issue. This issue may be solved by tuning the *ad hoc* noble metal(s)/support couple and adjusting the proper process conditions.

## Experimental

### Materials

Anhydrous glycerol 99%, from Sigma-Aldrich was used for the catalytic tests, and H_2_SO_4_ from Sigma Aldrich was used for HPLC analysis. Different supports were used for the synthesis of the catalysts: SiO_2_ MCM-41 from ACS Material and three oxide supports, gamma alumina, titanium oxide, and zeolite ZSM-5, all from Alfa Aesar. Potassium platinum (+IV) chloride and pure sodium hydroxide both from Sigma-Aldrich were used as precursors for the preparation of the Pt-supported catalysts, while sodium borohydride (>96% NaBH_4_) from Sigma-Aldrich was used as reducing agent and sodium hydroxide (NaOH ≥98%) from Sigma-Aldrich was used to adjust the pH.

### Catalysts Preparation

The platinum-based materials deposited on various supports (i.e., SiO_2_, TiO_2_, γ-Al_2_O_3_, ZSM-5) were prepared by chemical reduction in the liquid phase. Each powdered oxidic support (about 5 g) was suspended in 75 cm^3^ of ultrapure water and stirred for 60 min at 67°C. Then, a solution containing 0.3736 g K_2_PtCl_6_.6H_2_O (Sigma Aldrich, ≥98%) dissolved in 25 cm^3^ of ultrapure water was added dropwise to this suspension to obtain a platinum loading of about 1.5 wt.% relative to the support in the final material. The temperature of the reaction mixture was maintained at 67°C for 60 min under stirring. Sodium borohydride was used as a reducing agent to reduce the platinum metal. The amount of reducing agent was adjusted with respect to the amount of platinum precursor, each time using a 2-fold stoichiometric excess [11]. The as-obtained gray mixture was further stirred at 67°C, after adjusting the pH to 7 with a 0.3 M solution of sodium hydroxide. The suspension was then mixed at 67°C for 90 min and a gray solid was recovered by filtration, washed with 100 cm^3^ of distilled water and dried at 110°C for 24 h prior testing. A series of catalysts with a nominal content of platinum of 1.5 wt.% was then obtained.

### Catalysts′ Characterization

The textural properties (specific surface area, pore volume and mean pore size) of the catalysts were analyzed by N_2_ adsorption-desorption using a Micromeritics Tristar-II 3020 instrument. Prior to the analysis, the samples were outgassed at 110°C for 7 h under vacuum. To determine the Pt loading in the catalysts, inductively coupled plasma-atomic emission spectroscopy (ICP-AES, Vista Pro Varian) was used. The samples were prepared by dissolving the catalysts in a Hot Block/HCl/HNO_3_ digestion. The dispersion of Pt is determined by H_2_ chemisorption using Micromeritics Autochem II 2920 instrument. Prior to the analysis, pure H_2_ (i.e., 30 mL/min) was used to reduce the samples at 500°C (i.e., 10°C/min); Then an Ar flux (i.e., 50 mL/min) was used for 1 h at 500°C before a return to room temperature and 10 injections (20 μL) of H_2_ in Ar were carried out. The crystalline phases present in the catalysts were analyzed by XRD at ambient temperature on Bruker D8 Advance instrument, equipped with a CuKα source (λ = 0.154 nm). The samples were scanned at a rate of 0.02° over the 5° ≤ 2θ ≤ 90° range with an integration time of 0.5 s. The diffractograms were indexed using the JCPDS database. The oxidation state of platinum and its quantification on the surface of catalysts were determined by XPS analysis (AXIS Ultra Kratos) using a monochromatized aluminum source (AlKα = 1486.6 eV, 150 W), and the value of the C1s core level (285 eV) was used for the calibration of the energy scale. Curve fitting was performed using the Casa XPS software applying a Shirley-type background subtraction. To determine the size of the platinum particles, transmission electron microscopy analysis was used. TEM FEI Tecnai G2-20 twin, working with an accelerating potential of 200 kV and equipped with a slow-scan CCD camera, enabled the observation of samples with a very high resolution (2–5 nm scale). The samples for TEM were prepared from a diluted suspension of catalyst in ethanol. A drop of suspension was placed on a Lacey carbon-coated grid and allowed to dry in air. The particle size distribution was calculated by counting over 200 particles over multiple areas using the ImageJ software.

### Catalytic Performance Evaluation

The glycerol oxidation reaction in the liquid phase was carried out in a 300 cm^3^ semi-batch stainless steel reactor (PARR 5500 HP Series Compact Reactors), equipped with a thermocouple and a thermo-regulated oxygen supply system. For each experiment, 200 cm^3^ of an 0.1 M glycerol aqueous solution were heated to 80°C. The reaction was started at the temperature of 80°C when 0.5 g of the selected catalyst was introduced into the reactor immediately pressurized with 2 bar oxygen (*t*_0_). The products were periodically sampled and analyzed with an Agilent 1260 HPLC, equipped with an Aminex HPX-87H column (300 × 7.8 mm) and a reflective index detector (RID). A solution of H_2_SO_4_ (0.0025 M) in deionized water (flow rate: 0.5 cm^3.^min^−1^) was used as an eluent. The identification and quantification of the reaction products were performed using the corresponding calibration curves plotted upfront.

## Results and Discussion

### Catalysts Characterization

#### BET

The results of the textural analysis of the catalysts are presented in Table 1. Compared to supports alone, no change can be noted in terms of specific surface area for Pt/SiO_2_, Pt/TiO_2_, Pt/ZSM-5, and Pt/γ-Al_2_O_3_. The silica-supported catalyst (SiO_2_-MCM 41) exhibits the largest specific surface area (i.e., 625 m^2^/g) and the largest pore volume (0.575 cm^3^/g), but an average pore diameter of 2.7 nm. Pt/ZSM-5 and Pt/TiO_2_ have lower specific surface areas of 358 and 167 m^2^/g, respectively (Table 2). It can be noted that Pt/γ-Al_2_O_3_ has the lowest specific surface area (57 m^2^/g), but a higher pore diameter than the other studied materials (25.7 nm).

**Table 1:** List of the prepared catalysts with the corresponding acronyms, specific surface area (SSA_BET_), the BJH desorption branch-deduced average pore diameter (d_pores_) and the BJH desorption branch-derived cumulative pore volume (V_pores_).

**Catalyst**	**Pt loading** **(ICP)/Me wt.%**	**Pt dispersion** **(H_**2**_ chemisorption)/%**
Pt/SiO_2_	1.20	1
Pt/ZSM5	1.36	1.5
Pt/TiO_2_	1.15	24.5
Pt/γ-Al_2_O_3_	1.29	32.7

**Table 2:** List of catalysts prepared with the acronyms, the actual platinum metal loading estimated by elemental analysis (ICP), and dispersion estimated by H_2_-Chemisorption.

**Catalyst name**	**Support**		**Catalyst**	
	***SSA_BET_*** **(N_2_-BET)/m^2^.g^−1^**		***V_poresBJH_*/cm^3^.g^−1^**	***d_pores_*** **(nm)**
Pt/SiO_2_	635	625	0.575	2.7
Pt/ZSM-5	361	358	0.165	8.2
Pt/TiO_2_	173	167	0.396	6.5
Pt/γ-Al_2_O_3_	57	56	0.394	25.7

#### XRD

**Figure 1:**
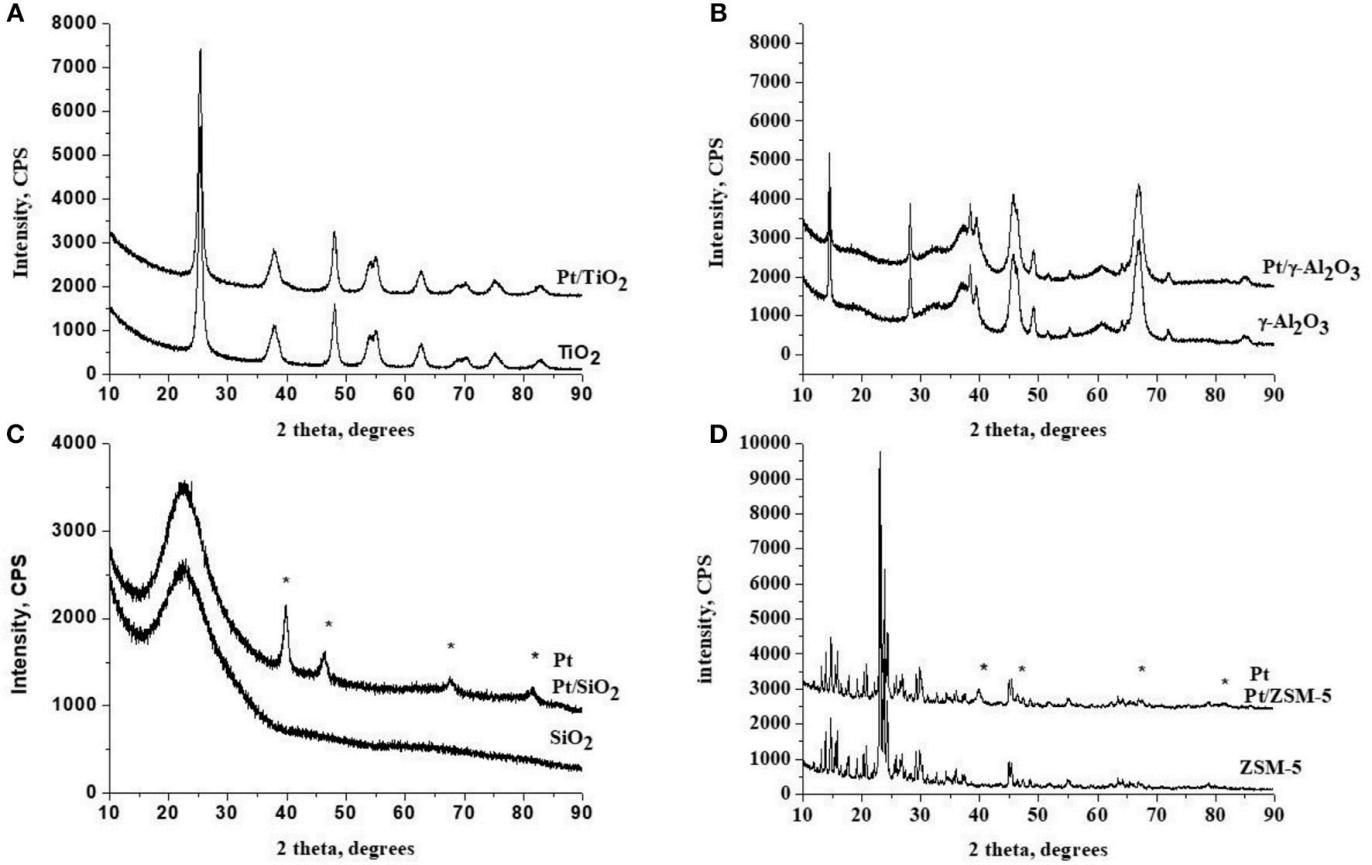
X-ray diffractograms of the fresh catalysts and of the corresponding supports with the position of the main diffraction lines expected for the metallic particles. Symbols (*) corresponds to: Pt. **(A)** TiO_2_ as support, **(B)** γ-Al_2_O_3_ as support, **(C)** SiO_2_ as support and **(D)** ZSM-5 as support.

The diffractograms of catalysts are presented in Figure 1. The diffractogram of the TiO_2_ support (Figure 1A) shows well resolved diffraction peaks, characteristic of the crystallized anatase phase in a tetragonal face-centered system (PDF 00-021-1272). There is no significant change in the diffractogram after platinum addition is observed. Figure 1B shows the diffractogram of the catalyst supported on gamma-alumina (PDF 001-1303). The diffractograms of the catalyst support are identical. The diffractogram of the SiO_2_ MCM-41 support (Figure 1C) has a very large and low intensity line representative of the amorphous character of this support. The appearance of several additional peaks on the Pt/SiO_2_ diffractogram is attributed to the presence of crystallized metallic platinum in a face-centered cubic lattice (JCPDS No. 04-0802). The diffractogram of the ZSM-5 support (Figure 1D) has well resolved diffraction peaks, characteristic of ZSM-5 zeolite. The appearance of additional peaks after Pt deposition (which are similar to those observed for silica MCM-41) is also attributed to the presence of crystallized metallic platinum (JCPDS No. 04-0802). According to the diffractogram analysis of the various catalysts, it can be concluded that the platinum supported on TiO_2_ and γ-Al_2_O_3_ is predominantly present under the form of small particles (with XRD, particles with a size <5 nm can be hardly identified). The clear appearance of characteristic diffraction peaks assigned to metallic platinum over SiO_2_ and ZSM-5 suggests the presence of Pt particles larger than 5 nm, which will be confirmed by TEM analysis (*vide supra*).

#### ICP & H_2_ Chemisorption

The results of the elemental analysis of the catalysts prepared shown in Table 1 confirm that the actual amount of deposited platinum is close to the theoretical one (i.e., 1.5 wt.%). Metal dispersions obtained by chemisorption of H_2_ are summarized in Table 1 as well. A very low platinum dispersion is obtained for Pt/SiO_2_ and Pt/ZSM-5 (1 and 1.5%, respectively). On the other hand, the platinum dispersion on TiO_2_ and γ-Al_2_O_3_ is much better, with respective values of 24.5 and 32.7%. These results suggest an agglomeration of the platinum particles on the two silicic supports. This is in agreement with the XRD analyzes results (i.e., detection of a metallic Pt phase suggesting particle sizes larger than 5 nm).

#### TEM

**Figure 2:**
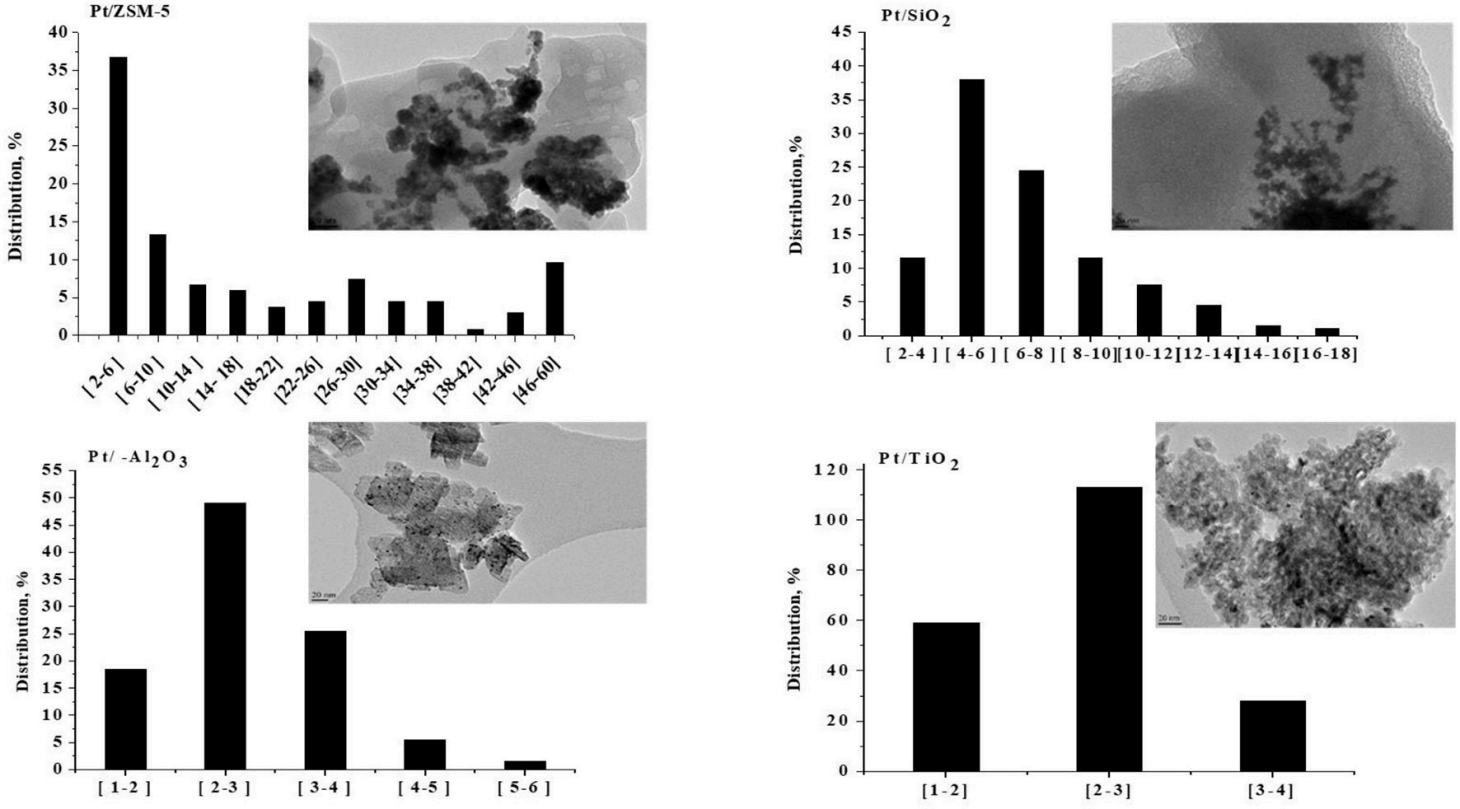
TEM images and histograms of the metallic particle size distributions observed over the prepared catalysts.

Figure 2 presents the TEM images of supported platinum-based catalysts, as well as the particle size distribution. A non-homogeneous distribution and aggregations of platinum particles were observed for Pt/SiO_2_. Over this sample, more than 50% of the particles have a size <6 nm, but the distribution extends to sizes larger than 18 nm. Note that we did not analyze all the agglomerated particles on the TEM pictures for that sample, which means that the actual number of very large agglomerates/particles is even underestimated. The TEM images of Pt/ZSM-5 catalyst show that the platinum particles' size distribution is not homogeneous over this sample with the presence of aggregates of platinum particles. One third of the population has a size between 2 and 6 nm, more than half is within the range of 6–36 nm and the remainder has a diameter larger than 36 nm. This agrees with the XRD observations as well. Concerning the Pt/TiO_2_ catalyst, the particle size distribution is centered on 2–3 nm with 55% of the particles in this range. The largest size observed is 4 nm. This result agrees with the observed metal dispersion (24.44%) and the absence of Pd peaks on the XRD diffractograms. Finally, The Pt/γ-Al_2_O catalyst has the best dispersion among all of the studied samples, with a particle size distribution centered on 2–3 nm, it gathered 50% of the total population. The largest size observed is 6 nm.

#### XPS

**Figure 3:**
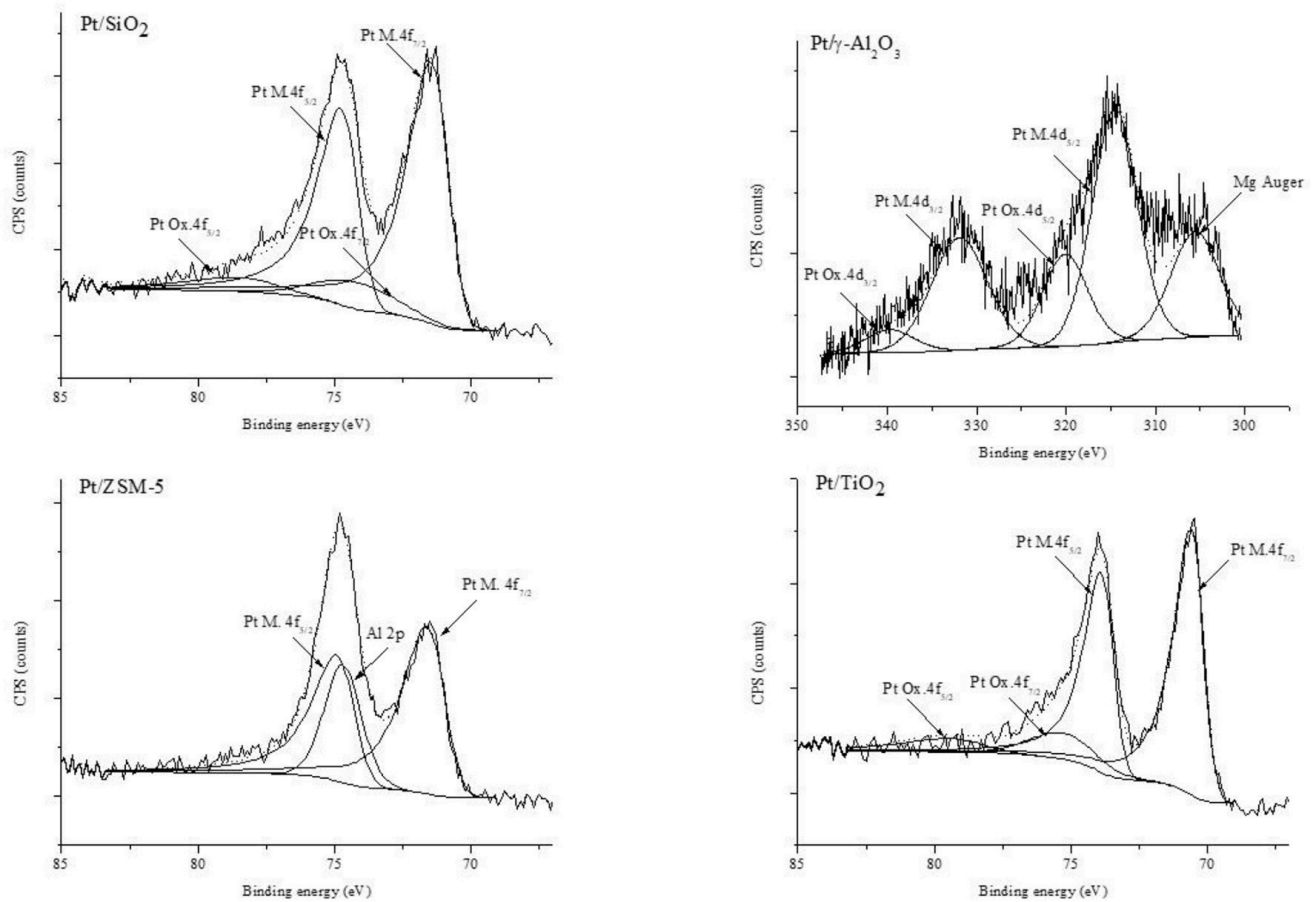
XPS spectra of the Pt 4d or Pt 4f levels with the corresponding BE values observed over the prepared catalysts.

The chemical surface composition of the samples has been studied by XPS. This study is based, for platinum, on the analysis of the Pt 4f levels for Pt/SiO_2_, Pt/TiO_2_, and Pt/ZSM-5 and of the Pt 4d level for Pt/γ-Al_2_O_3_. Indeed, for this latter, the Pt 4f photopeak overlaps that of Al 2p, which makes it unexploitable. It should be noted that the percentage of aluminum in the ZSM-5 support is lower than in the γ-Al_2_O_3_, and that it is therefore possible to separate the Pt 4f and Al 2p photopeaks for this sample. Figure 3 shows the corresponding spectra obtained for all the catalysts. The spectrum of Pt/TiO_2_ shows two photoelectron peaks at binding energies of 70.28 eV (Pt 4f_7/2_) and 73.58 eV (Pt 4f_5/2_). These binding energies correspond to those expected for Pt^O^ (Hu et al., 2010). The other two photoelectron peaks are located at binding energies of 75.30 eV (Pt 4f_7/2_) and 78.60 eV (Pt 4f_5/2_) and are characteristic of platinum in the oxidic form (Skrzynska et al., 2015; “X-ray Photoelectron Spectroscopy (XPS) Reference Pages, Element: Platinum, Home page: http://www.xpsfitting.com (access 15thSep. 2018).,” n.d.). We determined that platinum oxide represents about 15% of the total platinum Table 3. Concerning the Pt/ZSM-5 catalyst, two photoelectrons peaks characteristic of platinum metal, located at binding energies of 71.65 eV (Pt 4f_7/2_) and 74.95 eV (Pt 4f_5/2_), and a photoelectron peak corresponding to Al 2p level, located at a binding energy of 74.66 eV, could be observed. The platinum analyzed in this catalyst was therefore 100% in the metallic form (Pt^O^). As for Pt/SiO_2_, the spectrum obtained is composed of two photoelectron peaks characteristic of Pt^O^ at binding energies of 71.0 eV (Pt 4f_7/2_) and 74.33 eV (Pt 4f_5/2_) and two photoelectron peaks characteristic of platinum in the oxidic form, located at binding energies of 74.23 eV (Pt 4f_7/2_) and 77.53 eV (Pt 4f_5/2_). Regarding the Pt/γ-Al_2_O_3_ sample, two components characteristic of Pt in the metallic form (Pt 4d_5/2_ and Pt 4d_3/2_ localized at binding energies 314.55 and 331.93 eV, respectively) and two peaks characteristic of platinum in the oxidic form (at 320.08 and 339.72 eV, respectively), were observed. Furthermore, the presence of a photoelectron peak located at a binding energy of 305.04 eV was noted, corresponding to an impurity that could be magnesium (Mg Auger) present as impurity in one of the precursors used during the synthesis.

**Table 3:** Binding energies of the Pt 4f7/2 level for the Pt on SiO_2_, TiO_2_, ZSM-5 catalysts, and of the Pt 4d5/2 level for Pt/γ-Al_2_O_3_; Surface Pt/M atomic ratios with M = Si (2p), Ti (2p) ou Al (2p).

**Catalyst**	**Pt 4f_**7/2**_ metallic (eV)**	**Pt 4f_**7/2**_ oxide (eV)**	**Mol% Pt metallic**	**Mol% Pt oxide**	**Pt/M (atom/atom)**
Pt/SiO_2_	71.0	74.2	87	13	0.008
Pt/TiO_2_	70.3	75.3	85	15	0.022
Pt/ZSM-5	71.2	-	100	-	0.005
Pt/γ-Al_2_O_3_	**4d**_**5/2**_ 314.5	**4d**_**5/2**_ 320.0	76	24	0.014

*The bold text signifies that the XPS contribution was changed for this sample due to the fact that there was a significant overlap in 4f7/2 with the Al contribution*.

The results of the XPS analyses as well as the quantification of the different species are summarized in Table 3, below.

The Pt/M atomic ratios are larger for the Pt/TiO_2_ and Pt/γ-Al_2_O_3_, which can be explained by the better dispersion of Pt over these catalysts.

### Catalytic Performances

#### Evolution of Selectivity as a Function of Time

**Figure 4:**
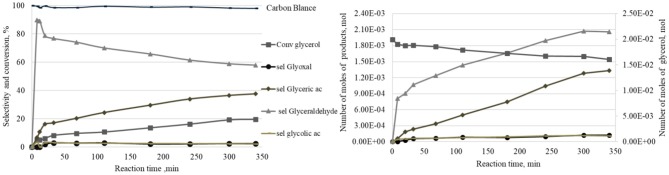
Evolution of the products quantities as a function of time under the following reaction conditions: 0.5 g of Pt/SiO_2_, 200 cm^3^ of an aqueous 0.1 M GLY solution, 2 bars of oxygen, 1,000 rpm, temperature 80°C.

The evolution of the quantity of the different products formed during a catalytic oxidation reaction of glycerol in liquid phase, using a Pt/SiO2 sample as an example under 0.1 M glycerol solution with 2 bars of oxygen at 80°C, was studied. As well as that, the consumption of glycerol as a function of time are shown in Figure 4 (right). Also, Figure 4 (left) presents the same data in the form of glycerol conversion and selectivities to products. At the beginning of the reaction, the oxidation of the primary hydroxyl group first leads to the formation of glyceraldehyde with a very rapid increase in the number of moles in the first 5 min with 13 mmol.mol^−1^Pt.h^−1^ as the initial formation rate. After 5 min, the rate of glyceraldehyde formation decreases. Further, the number of moles of glyceraldehyde remains stable after 300 min. On the other hand, glyceric acid appears after 8 min of reaction and its quantity progresses linearly. Two other minor products (i.e., glycolic acid and glyoxal) appear at the beginning of the reaction, without any subsequent evolution of their quantity with time. In terms of selectivity, Figure 4 shows that the glyceraldehyde selectivity reaches 90% after 8 min and then progressively decreases to 58% after 340 min. The selectivity to glyceric acid increases gradually to 38% after 340 min.

**Figure 5:**
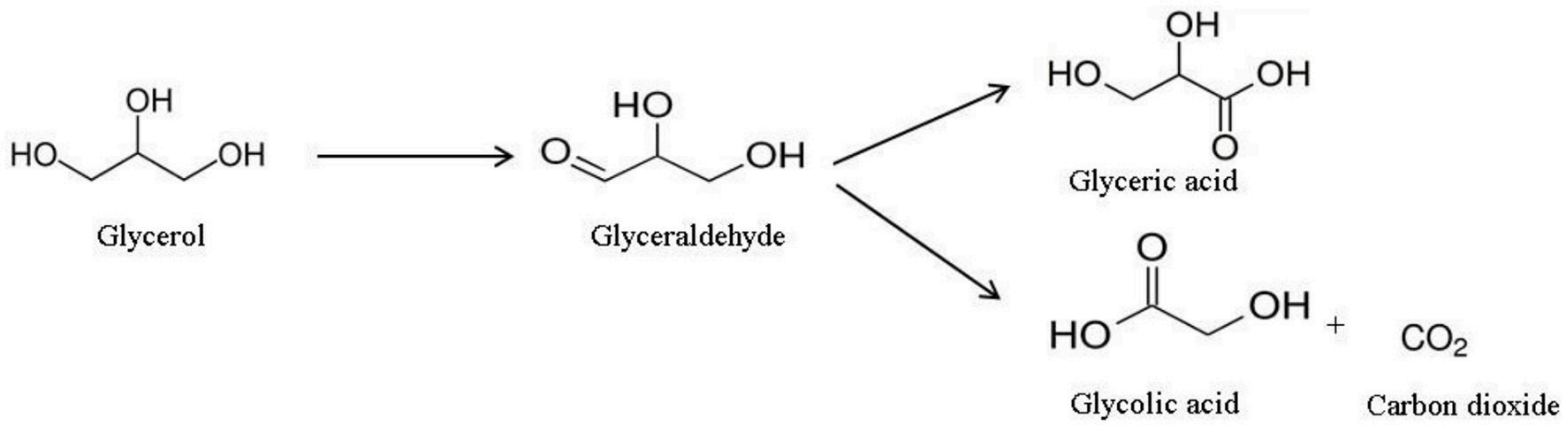
The proposed scheme of the oxidation of glycerol in base-free solution.

Owing to the obtained results, a reaction scheme (Figure 5) was proposed to explain the formation of the products (Katryniok et al., 2011). The first step of the reaction is the oxidation of the primary hydroxyl to form glyceraldehyde (primary product). The latter can be oxidized to glyceric acid or undergo oxidative C-C cleavage to form glycolic acid and carbon dioxide, as secondary products.

#### Influence of the Support

**Figure 6:**
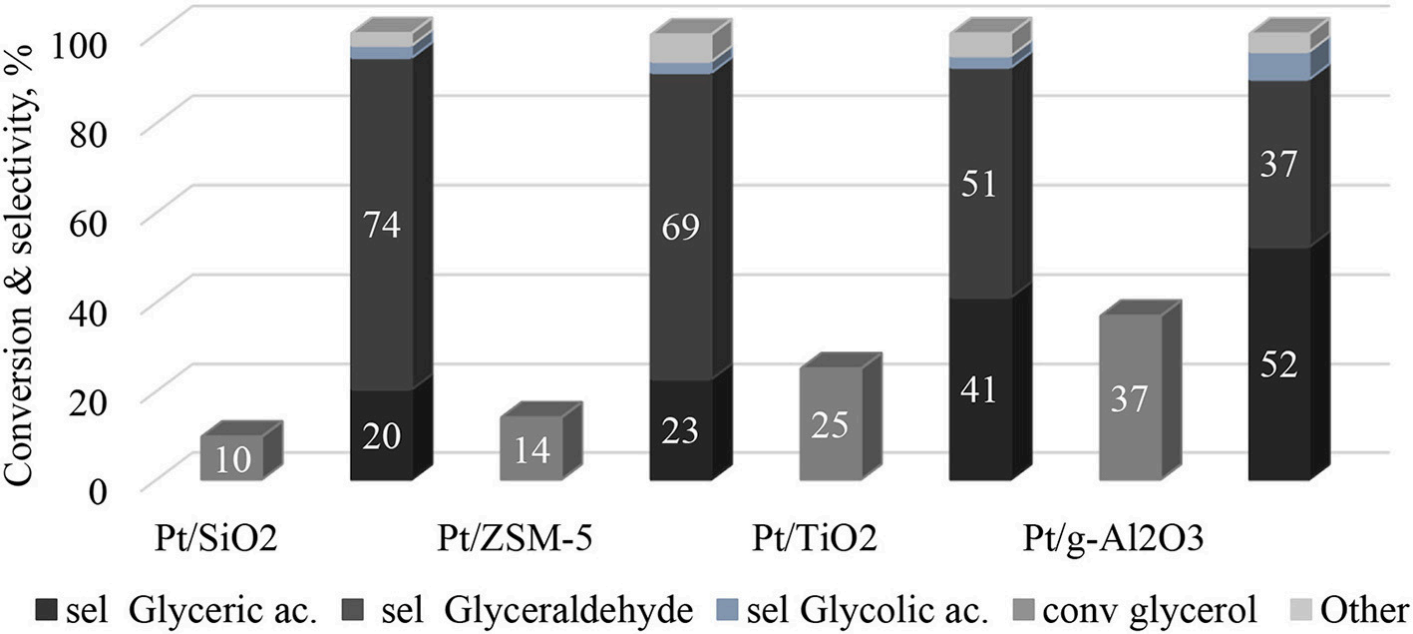
Conversion and selectivity of products during glycerol partial oxidation over the prepared catalysts. Reaction conditions: 0.5 g of catalysts, 200 cm^3^ of an aqueous 0.1 M GLY solution, 2 bars of oxygen, 1,000 rpm, temperature 80°C, 1 h.

The catalytic tests of the glycerol oxidation in the liquid phase were carried out for the four catalysts and the results are presented in Figure 6. The best performance is obtained with Pt/γ-Al_2_O_3_ with a glycerol conversion of 37% after 60 min of the reaction (with an initial glycerol conversion speed of 48 mmol glycerol.mol^−1^Pt.h^−1^). After the same reaction time, the Pt/TiO_2_ catalyst converts 25% of glycerol with an initial glycerol conversion rate of 52 mmol glycerol.mol^−1^Pt.h^−1^. Finally, the Pt/SiO_2_ and Pt/ZSM-5 catalysts are much less active with glycerol conversions of 10 and 14%, respectively, after 1 h (and initial glycerol conversion rates of 16 and 14 mmol glycerol.mol^−1^Pt.h^−1^, respectively). These differences can be interpreted according to the aforementioned characterization results. The analysis of the platinum dispersion showed that the platinum on SiO_2_ and on ZSM-5 is weakly dispersed, but well dispersed on TiO_2_ and γ-Al_2_O_3_. This result was confirmed by TEM analysis, which showed an agglomeration of platinum over SiO_2_ and ZSM-5. Therefore, this suggests that the number of active sites accessible on the Pt/SiO_2_ and Pt/ZSM-5 catalysts is lower than on the Pt/TiO_2_ and Pt/γ-Al_2_O_3_ catalysts. This result was further corroborated by XPS analysis, which showed that the atomic ratios (i.e., Pt/M, Atom/Atom) are lower for the Pt/SiO_2_ and Pt/ZSM-5 catalysts (Table 3)

**Figure 7:**
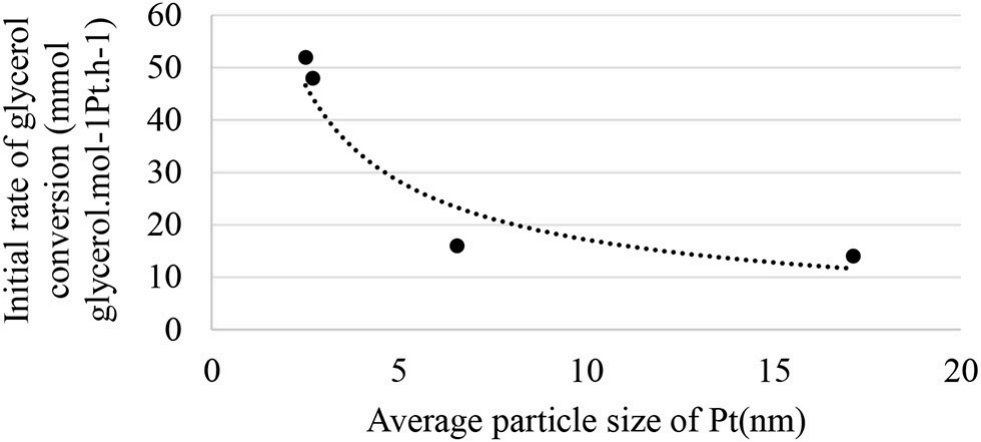
Relationship between the initial rate of glycerol conversion and the average particle size of Pt.

**Figure 8:**
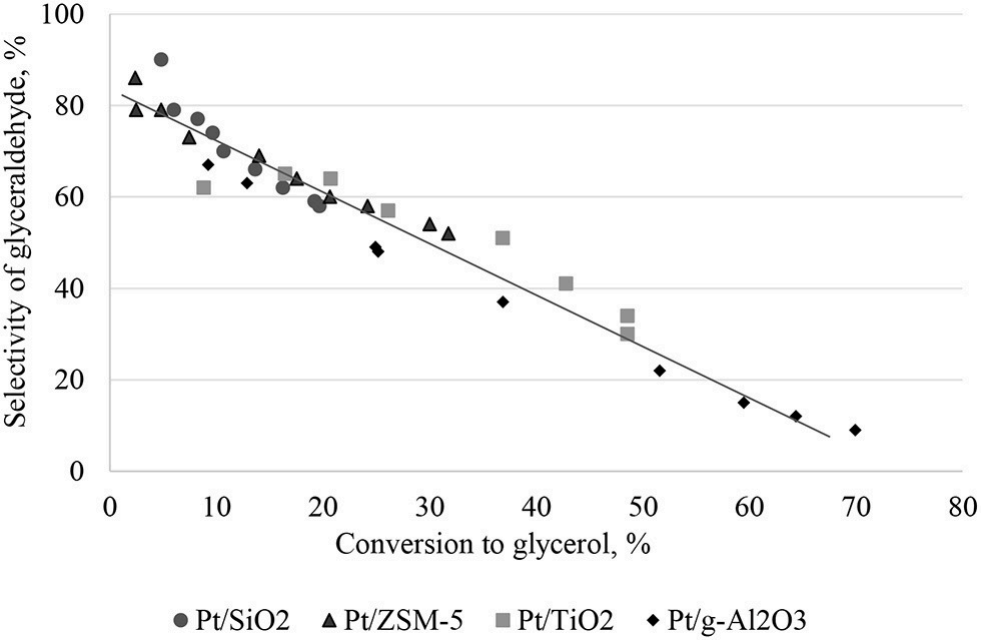
Relationship between the selectivity of glyceraldehyde and the conversion to glycerol.

Figure 7 shows the relationship between the initial rate of glycerol conversion and the average particle size of Pt. It is evident that from ~6 nm, the initial rate of transformation seems constant. According to Figure 6, the selectivity to glyceraldehyde (74%) is higher for the less active catalyst (Pt/SiO_2_-10% glycerol conversion and 20% glycerol acid selectivity after 60 min of reaction). The Pt/γ-Al_2_O_3_ catalyst is the most active in the series studied with a 37% glycerol conversion after 60 min of the reaction, predominantly producing glyceric acid (52%). It clearly exists a dependence between glyceraldehyde and glycerol acid selectivity and glycerol conversion, which is common for successive reactions of the A → B → C type. To interpret these results, the evolution of glyceraldehyde selectivity was plotted as a function of conversion for all of the catalysts. Figure 8 shows that the selectivity of the glyceraldehyde linearly decreases with the increase in conversion, and therefore the most active catalyst will have a low glyceraldehyde selectivity, which is the case for the Pt/Al_2_O_3_, Pt/TiO_2_, and Pt/ZSM-5.

## Conclusion

In this work, we synthesized four Pt based catalysts changing the support. XPS characterizations evidence that the platinum was mainly reduced but not totally for most used supports with a reduction level higher than 80%. The nature of the catalytic support clearly influences the platinum particle size and the dispersion of the particles has been demonstrated. The silicic supports studied generate aggregation of the platinum particles leading to a high average particle size (i.e., 6.5 nm) Contrary to that, using TiO_2_ or γ-Al_2_O_3_ as supports leads to very small particles (i.e., 2.7 nm) with a narrow size distribution. The average particle size leads to a significant change in the initial rates of glycerol oxidation in the liquid phase in a base-free solution. The catalyst having a low dispersion has a particle size distribution spread over a wide range and a larger average particle size (i.e., fewer active sites), inducing a lower glycerol conversion and initial transformation rate on these materials. We have also demonstrated a real dependence between target molecule selectivity (i.e., glyceraldehyde) and substrate conversion, where the selectivity of glyceraldehyde decreases with increasing glycerol conversion. At the same time, the oxidation of glyceraldehyde leads to the formation of glyceric acid, which does not seem to be transformed anymore in our reaction conditions. Thanks to our work, we have designed a series of catalysts able to transform glycerol either in glyceraldehyde or in glyceric acid in base free media, depending on material choice. In order to achieve high glyceraldehyde selectivity, low glycerol conversion is needed followed by a separation process. In this case, the Pt/SiO_2_ is a good candidate due to the fact that its initial transformation rate is lower allowing an easier separation process. If the goal is to obtain glyceric acid with high selectivity, Pt/γ-Al_2_O_3_ is much more suitable.

## Data Availability

All datasets generated for this study are included in the manuscript and/or the supplementary files.

## Author Contributions

AE: student who perform majority of the experiment; PF: contribution on the mechanism part; FD: contribution on the characterization part (XPS,…); MC: contribution on the reactivity part. All authors have contributed to the manuscript writing process.

### Conflict of Interest Statement

The authors declare that the research was conducted in the absence of any commercial or financial relationships that could be construed as a potential conflict of interest.
